# IFN-****γ****-Secreting-Mesenchymal Stem Cells Exert an Antitumor Effect *In Vivo* via the TRAIL Pathway

**DOI:** 10.1155/2014/318098

**Published:** 2014-05-26

**Authors:** Xinyuan Yang, Jingchun Du, Xia Xu, Chun Xu, Wu Song

**Affiliations:** ^1^Department of Gynecology and Obstetrics, First Affiliated Hospital, School of Medicine, Xi'an Jiaotong University, Xi'an 710061, China; ^2^Kingmed College of Laboratory Medicine, Guangzhou Medical University, Guangzhou 510182, China; ^3^Department of Gastrointestinal-Pancreatic Surgery, First Affiliated Hospital of Sun Yat-Sen University, Guangzhou 51800, China

## Abstract

Mesenchymal stem cells (MSCs) can exhibit either prooncogenic or antitumor properties depending on the context. Based on our previous study, we hypothesized that MSCs engineered to deliver IFN-*γ* would kill cancer cells through persistent activation of the TRAIL pathway. Human bone-marrow (BM-) derived MSCs were isolated, amplified, and transduced with a lentiviral vector encoding the IFN-*γ* gene under the control of the EF1*α* promoter. The IFN-*γ*-modified MSCs effectively secreted functional IFN-*γ*, which led to long-term expression of TRAIL. More importantly, the IFN-*γ*-modified MSCs selectively induced apoptosis in lung tumor cells through caspase-3 activation within the target cells. The percentage of activated-caspase-3-positive tumor cells in IFN-*γ*-modified MSCs cocultures was significantly higher than in control MSCs cocultures. Treatment with anti-TRAIL antibody dramatically suppressed the caspase-3 activation observed in H460 cells. After injection into nude mice, the IFN-*γ*-modified MSCs inhibited the growth and progression of lung carcinoma compared with control cells. Collectively, our results provide a new strategy for tumor therapy that utilizes IFN-*γ*-modified MSCs.

## 1. Introduction

Mesenchymal stem cells (MSCs) are derived from the mesodermal germ layer and can be easily isolated and cultured from many tissues, such as bone marrow, adipose tissue, and amniotic fluid [1–3]. Because MSCs exhibit low expression of MHC I and do not express MHC II molecules, these cells do not cause immunological reaction and rejection after they are infused into an allogenic body [4, 5]. In addition, other reports have indicated that MSCs exhibit tropism toward tumors [6]. Therefore, MSCs are considered a promising cellular carrier for tumor gene therapy. For example, IFN-*α*-engineered MSCs can halt tumor growth* in vivo* by activating immune cells [7], and MSCs modified with pigment epithelium-derived factor can inhibit hepatocellular carcinoma growth* in vivo* by decreasing angiogenesis [8].

In addition to isolation from normal tissue, MSCs have also been found in tumor tissue and may be a key source of tumor-associated stromal cells [9, 10]. The current evidence suggests that the role of MSCs in tumor survival and progression is complex and diverse [11]. In our previous report, IFN-*γ*-primed MSCs were shown to express tumor necrosis factor-related apoptosis-inducing ligand (TRAIL), which causes apoptosis in tumor cells [12]. However, the expression of TRAIL only persisted for approximately seventy-two hours after one priming with IFN-*γ*. We therefore evaluated whether IFN-*γ*-secreting mesenchymal stem cells could exert a persistent antitumor effect via the TRAIL pathway.

In this study, we show that IFN-*γ*-modified MSCs continuously express TRAIL and induce apoptosis in lung cancer cells by activating caspase-3 within the target cells.* In vivo* models further confirm the antitumor effect of the IFN-*γ*-secreting MSCs.

## 2. Materials and Methods

### 2.1. Isolation, Expansion, and Differentiation of MSCs

According to a policy approved by our institutional ethics committee, heparinized human bone marrow (BM) samples were obtained by iliac crest aspiration from healthy donors who had given informed consent. MSCs were isolated and cultured as previously described [12]. Briefly, BM aspirates were diluted with an equal volume of low-glucose, complete DMEM and then fractionated with Lymphoprep solution (Huajin, Shanghai, China). The mononuclear cells were collected, washed, plated in 75 cm^2^ flasks, and incubated at 37°C under 5% CO_2_. After 3 days of culture, the nonadherent cells were removed by replacing the medium, and the adherent cells were continuously cultured with a change of the medium every 3 days. The MSCs were digested and replated when they reached approximately 90% confluence.

Osteogenic differentiation of the MSCs was induced using low-glucose, complete DMEM supplemented with 0.1 *μ*M dexamethasone (Sigma-Aldrich, St. Louis, MO), 50 *μ*g/L vitamin C (Sigma-Aldrich), and 10 mM *β*-glycerophosphate (Calbiochem, San Diego, CA). The medium was replaced every 2-3 days. Around day 15, the mineralization of the MSCs was assessed by staining with 0.5% alizarin red solution.

Adipogenic differentiation of the MSCs was performed as follows. First, the MSCs were incubated in adipogenesis-inducing medium (AIM) containing 1 *μ*M dexamethasone, 0.2 *μ*M indomethacin (Sigma-Aldrich), 0.5 mM IBMX (Sigma-Aldrich), 0.01 mg/mL insulin (Sigma-Aldrich), and 10% (v/v) FBS in high-glucose DMEM. After 72 hours, the AIM was replaced by adipogenesis-maintenance medium containing 0.01 mg/mL insulin (Sigma-Aldrich) and 10% (v/v) FBS in high-glucose DMEM; the cultures were maintained for 24 hours. This cycle was repeated 3 times. At day 13, the adipogenesis of the MSCs was assessed by staining with fresh 0.25% oil red O solution.

Chondrogenic differentiation of the MSCs was induced using a cell pellet culture system [13]. Briefly, 5 × 10^5^ MSCs were suspended in 2 mL high-glucose, complete DMEM supplemented with 10 ng/mL TGF-*β*1 (PeproTech, Rocky Hill, NJ), 6.25 *μ*g/mL insulin, 6.25 *μ*g/mL transferrin, 1 mM sodium pyruvate (Sigma-Aldrich), 37.5 *μ*g/mL ascorbate-2-phosphate, and 0.1 *μ*M dexamethasone and centrifuged at 500 g for 5 min in a 15 mL conical tube. The conical tube containing the compressed cells was placed in an incubator with a loosened cap. The medium was replaced every 3 days for 21 days. The chondrogenesis of the MSCs was assessed by staining with 1% alcian blue solution.

### 2.2. Construction of Lentiviral Vectors

The sequence encoding human IFN-*γ* (Funeng, Guangzhou, China) was first amplified using primer 1 (5′-GGGG ACA AGT TTG TAC AAA GCA GGCT GCC ACC ATG AAA TAT ACA AGT TAT ATC TTG GCT-TTT3′) and primer 2 (5′-GGGG ACC ACT TTG TAC AAG AAA GCT GGG T TTA CTG GGA TGC TCT TCG ACC TC-3′). After gel-purification, the PCR products were cloned into pDONR 221 through a BP recombination reaction following the manufacturer's instructions (Invitrogen, Carlsbad, CA) to generate the entry clone pDown-IFN-*γ*. Then, pUp-EF1*α* and pDown-IFN-*γ* were recombined into pDESTpuro through an LR recombination reaction to construct the expression vector pLV/Final-puro-EF1*α*-IFN-*γ*. Finally, PCR and sequencing were used to identify clones with the correct recombination events.

### 2.3. Lentivirus Construction and Transfection

The lentiviral particles carrying the IFN-*γ* or humanized Renilla GFP genes were prepared by transient cotransfection of pLV/Final-puro-EF1*α*-IFN-*γ* or pLV/Final-puro-EF1*α*-hrGFP with a lentiviral packaging mix (Invitrogen) into 293FT cells using Lipofectamine 2000 (Invitrogen) according to the manufacturer's instructions. After 48 hours, the viral particles were harvested, filtered through a 0.45 *μ*m polyethersulfone membrane, and concentrated by ultracentrifugation. Human MSCs were transduced with the lentiviral particles carrying IFN-*γ* or hrGFP at a multiplicity of infection of 50. At the same time, the 3T3, 293FT, H460, H1299, A549, and MCF-7 cell lines were transduced with lentiviral particles carrying only hrGFP using the same protocol. After two rounds of infection, 1–5 *μ*g/mL puromycin was added to the culture medium, and these cultures were maintained for 2-3 days. The isolated cell lines were defined as MSCs IFN-*γ*, MSCs hrGFP, 3T3 hrGFP, 293FT hrGFP, H460 hrGFP, H1299 hrGFP, A549 hrGFP, and MCF-7 hrGFP, respectively.

### 2.4. Real-Time PCR

Total RNAs were extracted from undifferentiated and differentiated MSCs using TRIzol reagent according to the manufacturer's instructions (Invitrogen). After digestion with DNase I (Fermentas, Burlington, Canada), 1 ug total RNA was reverse-transcribed using RevertAid first strand complementary DNA synthesis kit (Fermentas). All quantitative real-time PCR assays were performed on CFX Connect Detection System (Bio-Rad, Hercules, CA, USA) using SYBR Green I Master Mix (TOYOBO, Osaka, Japan). All reactions were run in triplicate. Relative quantification (RQ) was performed by normalizing the expression of target gene to that of GAPDH, used as a reference. The minimally normalized detectable target gene expression level was assigned a value of unity, and the other values reflect fold changes compared with this minimal level. The primer sequences used in this study were as follows: osteocalcin: 5′-CAC TCC TCG CCC TAT TGG C-3′ and 5′-CCC TCC TGC TTG GAC ACA AAG-3′; *α*P2, 5′-AGC ACC ATA ACC TTA GAT G-3′ and 5′-CGT GGA AGT GAC GCC TTT C-3′; collagen I: 5′-CAG CCG CTT CAC CTA CAG C-3′ and 5′-TGT ATT CAA TCA CTG TCT TGC-3′; and GAPDH: 5′-GAA GGT GAA GGT CGG AGT C-3′ and 5′-GAA GAT GGT GAT GGG ATT TC-3′.

### 2.5. ELISA

The supernatant media derived from the control MSCs, MSCs IFN-*γ*, and MSCs hrGFP were collected, and the IFN-*γ* in these media was measured using a human IFN-*γ* ELISA kit (eBioscience, San Diego, CA) according to the manufacturer's instructions.

### 2.6. Western Blots (WB)

MSCs were first primed with IFN-*γ* (20 ng/mL). After 12 hours, the medium was replaced with normal low-glucose complete DMEM, and the cultures were maintained for 72 hours. Thereafter, the MSCs were repeatedly primed with IFN-*γ* for 12 hours and then again maintained in normal medium for 24 hours. At different time points, the MSCs were washed with cold PBS and lysed in Laemmli buffer. The cell lysates were denatured at 100°C for 5 min and centrifuged at 12,000 g for 10 min at 4°C. The supernatants were recovered, separated by 12% SDS-PAGE, and transferred onto 0.45 *μ*m PVDF membranes (Millipore, Bedford, MA). After blocking with TBS-Tween-20 (0.1%) containing 5% nonfat milk for 1 hour at room temperature, the PVDF membranes were incubated with the appropriate primary antibodies (anti-TRAIL or anti-GAPDH antibody) overnight at 4°C. Specifically bound primary antibodies were detected by peroxidase-coupled secondary antibodies and chemiluminescence (Cell Signaling Technologies, Beverly, MA). In addition, the expression of TRAIL in MSCs IFN-*γ* was also evaluated using this method.

### 2.7. Coculture Experiments

MSCs IFN-*γ* or MSCs were preplated in six-well plates at a density of 1 × 10^5^ cells per well and incubated overnight. Then, 3T3 hrGFP (4 × 10^5^), 293FT hrGFP (4 × 10^5^), H460 hrGFP (4 × 10^5^), H1299 hrGFP (4 × 10^5^), A549 hrGFP (4 × 10^5^), or MCF-7 hrGFP (4 × 10^5^) cells were added to the wells. After 48 hours of coculture, the apoptotic level and percentage of green fluorescent cells were assessed by microscopic analysis after staining with DAPI (Sigma-Aldrich), and the total cell viability in the MSC IFN-*γ* cocultures was evaluated using a CCK-8 kit (Dojindo, Japan). These characteristics are reported as a percentage normalized to the values of the control MSC cocultures, which were set to 100%. Additionally, the levels of activated caspase-3 in the H460 hrGFP cells were measured using an antiactive caspase-3 antibody (Promega, Madison, WI) at different time points during coculture.

### 2.8. Xenograft Cancer Models

Six-week-old athymic nude mice were purchased from the Guangdong Medical Laboratory Animal Center and used in accordance with institutional guidelines under approved protocols. A total of 1 × 10^6^ H460 cells combined with or without 3 × 10^5^ MSCs or MSCs IFN-*γ* were suspended in 100 *μ*L of PBS and subcutaneously injected into the flank region of nude mice. On the fourteenth day after the first injection, the same numbers of MSCs or MSCs IFN-*γ* were again injected at the same position. The mice were examined three times a week, and the sizes of the tumors were calculated as reported [14]: volume = length × width^2^/2. After 60 days, the tumor masses were excised after the mice were sacrificed, and the tumors were removed, dissected, and characterized by hematoxylin and eosin staining.

### 2.9. Statistics

The data are expressed as the means ± SD. A two-tailed *P* value less than 0.05 from a Student *t*-test performed using SPSS version 12.0 (SPSS Inc., Chicago, IL) was considered statistically significant.

## 3. Results

### 3.1. Characterization of MSCs and TRAIL Expression Pattern after IFN-*γ*-Priming

**Figure 1:**
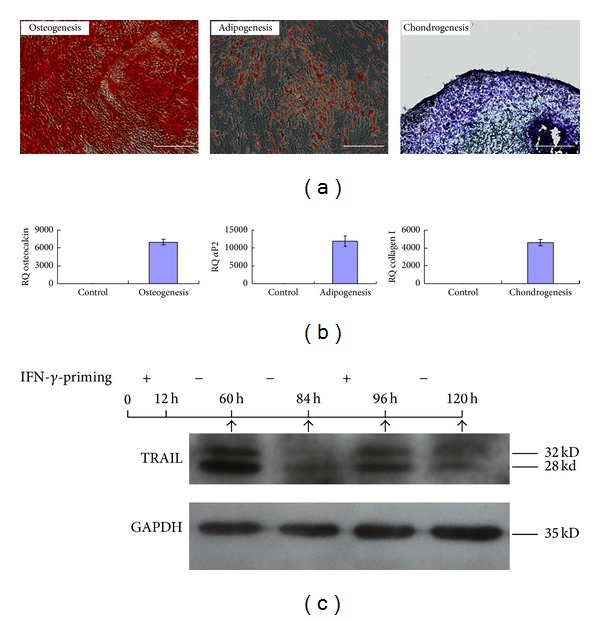
The characteristics of MSCs were analyzed. (a) The osteogenic, adipogenic, and chondrogenic differentiation of MSCs were identified by alizarin red S staining, oil red O staining, and alcian blue staining, respectively. Scale bar: 200 *μ*m. (b) After 7 days of induction, the expression of genes involved in osteogenic, adipogenic, and chondrogenic differentiation was measured using quantitative real-time PCR and normalized to GAPDH expression. (c) TRAIL expression in MSCs undergoing IFN-*γ*-priming was analyzed by Western blotting. IFN-*γ*-primed MSCs showed reduced expression of TRAIL after the removal of IFN-*γ* but continuously expressed TRAIL when stimulated again with IFN-*γ*.

First, the characteristics of MSCs were evaluated using differentiation assays. As shown in Figures 1(a)-1(b), MSCs could easily differentiate into osteoblasts, adipocytes, and chondrocytes under suitable induction conditions.

In addition, the TRAIL expression in MSCs only lasted approximately 72 hours after one IFN-*γ*-priming. However, these MSCs continued to express TRAIL if they were primed again with IFN-*γ* (Figure 1(c)).

### 3.2. MSCs Could Be Genetically Modified with IFN-*γ* to Express TRAIL

**Figure 2:**
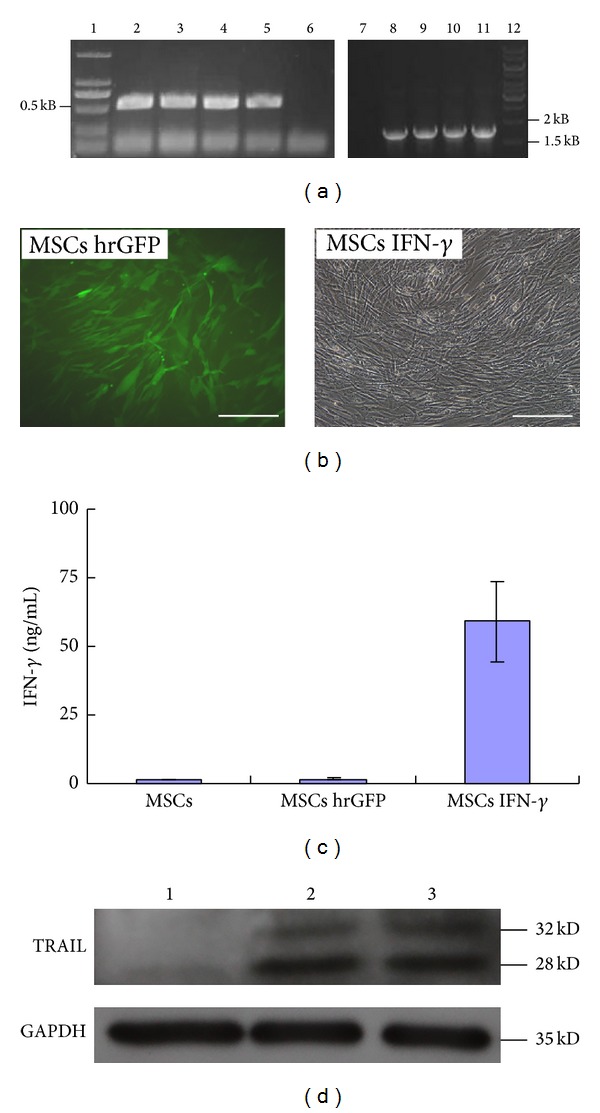
Construction of IFN-*γ*-modified MSCs. (a) The lentiviral vector pLV/Final-puro-EF1*α*- IFN-*γ* was identified by PCR. Lane 6/7: negative controls; lanes 2–5: amplification fragments of the IFN-*γ* coding sequence; lanes 8–11: amplification fragments of the EF1*α* promoter sequence. (b) The morphology of MSCs hrGFP and MSCs IFN-*γ*. Scale bar: 200 *μ*m. (c) The expression of IFN-*γ* was assayed by ELISA. (d) TRAIL expression in MSCs IFN-*γ* was determined by Western blotting analysis. Lane 1: negative control; lane 2/3: MSCs IFN-*γ* at P2 and P5 passages.

The lentiviral expression vector pLV/Final-puro-EF1*α*-IFN-*γ* was constructed using multisite gateway technology and then confirmed by PCR (Figure 2(a)) and DNA sequencing (data not shown). Lentivirus particles containing the IFN-*γ* or hrGFP genes were constructed in 293FT cells. MSCs were then transduced with the lentiviral particles. After puromycin selection, more than 90% of the MSCs were hrGFP-modified (Figure 2(b)). Because the transductions were performed in parallel, it could be inferred that the transduction efficiencies were similar to both the IFN-*γ* virus and the hrGFP virus. Moreover, ELISA results indicated that MSCs IFN-*γ* efficiently secreted IFN-*γ* (Figure 2(c)). Western blot results indicated that MSCs IFN-*γ* synthesized TRAIL at P2 and P5 passages, respectively (Figure 2(d)). On the other hand, the immunophenotype, differentiation potential, and proliferation property of MSCs IFN-*γ* were not dramatically changed, compared with those of MSCs (see Figure S1 in the Supplementary Material available online at http://dx.doi.org/10.1155/2014/318098).

### 3.3. IFN-*γ*-Secreting MSCs Selectively Induce Apoptosis in Tumor Cells* In Vitro*

**Figure 3:**
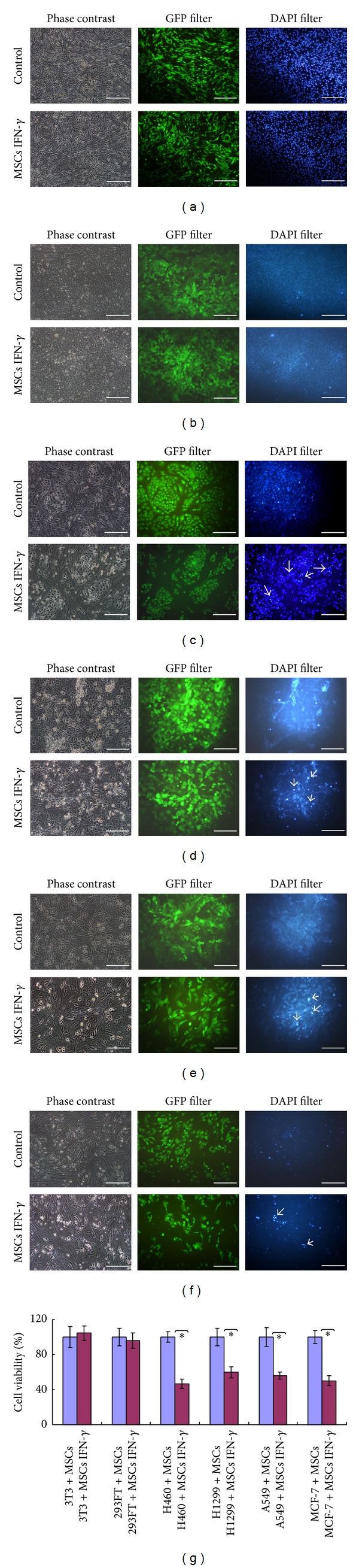
MSCs IFN-*γ* selectively induced apoptosis in cancer cells, but not in normal cells. MSCs IFN-*γ* were cocultured with 3T3 hrGFP (a), 293FT hrGFP (b), H460 hrGFP (c), H1299 hrGFP (d), A549 hrGFP (e), and MCF-7 hrGFP (f) cells for 48 hours. The mixed cells were observed using phase-contrast, GFP-specific, and DAPI-specific microscopy. The typical apoptotic and dead cells are marked. Scale bar: 200 *μ*m. (g) The cell viability within the coculture system was measured using a CCK-8 kit and was shown as a percentage normalized to the viability of the control MSCs coculture group, which was set to 100%. The asterisks indicate *P* < 0.01. The reported results are representative of the results from three separate experiments.

As shown in Figures 3(a)–3(f), MSCs IFN-*γ* induced apoptosis in H460, H1299, A549, and MCF-7 cancer cells, but not in 3T3 and 293FT cells; apoptosis was evident by the detachment of GFP-positive target cells and the appearance of cellular debris and apoptotic bodies in GFP-positive cells. The observed cell viability, as detected by the CCK-8 kit, also indicated that MSCs IFN-*γ* inhibited the proliferation of the transformed tumor cells to a greater extent than control MSCs (*P* < 0.05) (Figure 3(g)).

### 3.4. IFN-*γ*-Secreting MSCs Kill Tumor Cells by TRAIL-Mediated Caspase-3 Activation

**Figure 4:**
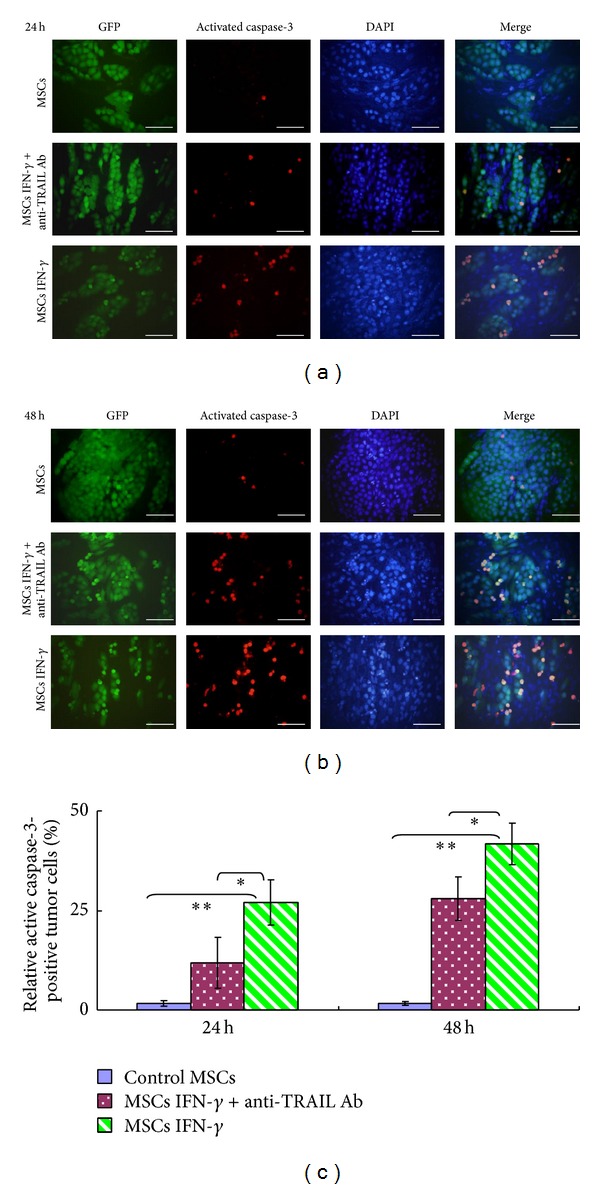
MSCs IFN-*γ* induced tumor cell apoptosis by activating caspase-3 within the target cells, a process which was mediated by TRAIL. MSCs IFN-*γ* were cocultured with H460 hrGFP cells for 24 h (a) and 48 h (b) in the presence or absence of an anti-TRAIL antibody. The activated caspase-3 was detected in the H460 hrGFP cells by immunofluorescence staining. (c) The percentage of activated caspase-3-positive H460 hrGFP cells was quantified using ImagePro 5.0 (*P < 0.05, **P < 0.01). The reported results are representative of the results from three separate experiments.

To further explore the mechanisms underlying MSCs IFN-*γ*-induced apoptosis, the activation of caspase-3 within target cells was measured. As shown in Figure 4, the percentage of H460 cells positive for activated caspase-3 was significantly higher for those cocultured with MSCs IFN-*γ* than those cocultured with control MSCs (27.1 ± 5.6% at 24 h and 41.7 ± 5.0% at 48 h compared with 1.6 ± 0.8% at 24 h and 1.6 ± 0.5% at 48 h, resp., *P* < 0.01). However, treatment with an anti-TRAIL antibody dramatically suppressed the observed caspase-3 activation in H460 cells (11.8 ± 6% at 24 h and 27.9 ± 5.7% at 48 h, *P* < 0.05). These results indicate that MSCs IFN-*γ* effectively induce apoptosis in tumor cells by activating caspase-3 within the target cells and reveal the TRAIL-mediated cytotoxic effect of MSCs IFN-*γ*.

### 3.5. IFN-*γ*-Secreting MSCs Exhibit Antitumor Activity* In Vivo*

**Figure 5:**
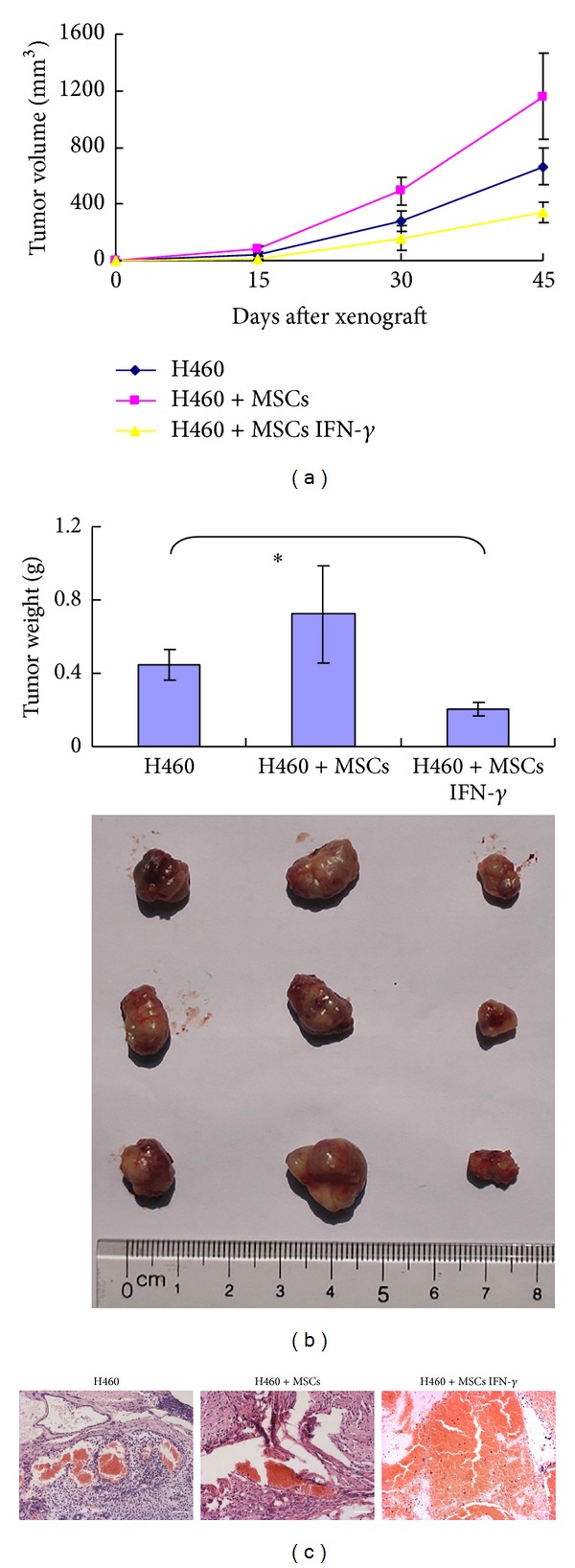
MSCs IFN-*γ* inhibit tumor mass growth in nude mice. (a) Tumor sizes were dynamically monitored after H460 cells combined with or without MSCs or MSCs IFN-*γ* were injected into nude mice (*n* = 3 mice/group, three separate experiments). (b) The tumor mass weight and morphology were compared at the end of the incubation period* in vivo*. The asterisks indicate *P* < 0.05. (c) Hematoxylin and eosin (H&E) staining of xenograft tumor. Tumor mass containing MSCs IFN-*γ* showed a serious extent of necrotic area, compared with the other two groups.

To evaluate the cytotoxicity of MSCs IFN-*γ in vivo*, xenograft tumor model of lung carcinoma was established, and the kinetics of tumor mass growth in nude mice were recorded. As shown in Figure 5(a), at approximately 20 days, tumor masses were observed in mice injected with H460 cells alone. The inclusion of MSCs IFN-*γ* delayed the appearance of tumors and inhibited the growth of the tumor mass. Finally, the tumor masses among the three groups had significantly different weights (0.45 ± 0.09 g in the H460 group, 0.21 ± 0.04 g in the MSCs IFN-*γ* group, and 0.72 ± 0.26 g in the MSCs group, *P* < 0.05). Correspondingly, hematoxylin and eosin staining indicated that tumor derived from the mixture of H460 and MSCs IFN-*γ* displayed dramatically tissue necrosis such as hemorrhagic region and fractured nuclei, compared with the other two groups (Figures 5(b)-5(c)).

## 4. Discussion

As mesoderm-derived progenitor cells, MSCs can home to tumor tissues through the chemotactic action of inflammatory factors [15]. However, the effects of MSCs on the growth and progression of tumors are still debated. For example, Kidd et al. reported that BM- and adipose-derived MSCs may be recruited by ovarian and breast tumors, are induced to become tumor-associated fibroblasts and vascular stromal cells, and promote tumor progression [16]. Luo et al. reported that BM-derived MSCs could promote prostate cancer metastasis via alteration of the CCL5-AR signaling pathway [15]. On the other hand, Zhu et al. reported that MSCs could inhibit K562 cell growth by secreting DKK1 [17], and Sun et al. reported that umbilical cord blood and adipose-derived MSCs could reduce lung metastasis and the growth of breast cancer cells by inducing apoptosis [18]. These results suggest that the effect of MSCs on tumors is variable and determined by the distinct tumor microenvironment and tumor type.

Alternatively, MSCs could produce variable responses under different induction conditions. For example, TLR4-primed MSCs have an antitumor effect, whereas TLR3-primed MSCs promote tumor growth and metastasis [19, 20]. Our previous report showed that IFN-*γ* induces MSCs to express TRAIL, which selectively mediates the apoptosis of tumor cells* in vitro*. However, the* in vivo* effect of IFN-*γ*-primed MSCs on tumor growth is different than that observed* in vitro* [12]. A primary reason for this discrepancy may be that the MSCs stimulated only once with IFN-*γ* could not maintain TRAIL expression, weakening the cytotoxic effect of the IFN-*γ*-primed MSCs. Here, the assumption that TRAIL expression is reduced in IFN-*γ*-primed MSCs after the removal of IFN-*γ* was confirmed. However, these MSCs continued to express TRAIL if IFN-*γ* was again supplemented.

As a type II interferon, IFN-*γ* is mainly produced by lymphocytes and NK cells and plays an important role in the adaptive cellular immune response against tumors. In addition, because the IFN-*γ* receptor is ubiquitously expressed, IFN-*γ* can also influence a vast number of nonlymphoid cellular responses by upregulating the expression of a number of apoptosis-associated proteins [21, 22]. IFN-*γ* has been considered a promising antitumor drug. However, the clinical application of IFN-*γ* in protein form is hindered by serious side effects, which result from the high dose required to overcome the short half-life of the protein and to achieve significant therapeutic effects [23, 24].

Based on the work presented here, the benefits of IFN-*γ* may be exploited through the genetic engineering of MSCs, which are used as a cellular vector. In addition to their function as a cellular vector, MSCs also exhibit a cytotoxic response after priming with IFN-*γ*. To achieve the ideal transduction efficiency, a lentiviral vector system was chosen for our experiments. Compared to other genetic engineering systems, such as adenoviral or retroviral vectors, the HIV-based lentiviral vector system can stably transfect cells at different mitotic stages, which is critical for MSCs that are often quiescent [25, 26]. Several studies have reported that lentiviral vectors can effectively deliver target genes into MSCs [8, 27]. Correspondingly, our results also indicated that the IFN-*γ* gene could be stably inserted into MSCs, expressed, and secreted by the host cells using a lentiviral vector. At the same time, IFN-*γ* could induce MSCs to stably synthesize TRAIL in an autocrine or paracrine fashion.

Most importantly, our data indicate that IFN-*γ*-secreting MSCs exert a selective cytotoxic effect on different types of tumor cell lines, including lung cancer cells and breast cancer cells. This cytotoxicity of IFN-*γ*-modified MSCs is mediated by TRAIL via activation of the extrinsic apoptosis pathway. After injection into nude mice along with H460 tumor cells, IFN-*γ*-modified MSCs still maintained an antiproliferative effect. The antitumor effect of IFN-*γ*-modified MSCs is obviously enhanced compared with that of IFN-*γ*-primed MSCs, as previously reported [12]. However, similar to our report, this study showed that tumor growth in mice injected with H460 cells was more prominent when MSCs were included [12]. A possible reason for this phenomenon may be that the proapoptotic capacity of IFN-*γ*-modified MSCs is dominant over the tumor-supportive capacity of unmodified MSCs, resulting in the inhibition of tumors by IFN-*γ*-modified MSCs.

In conclusion, our data indicate that MSCs can be effectively modified with the IFN-*γ* gene using a lentiviral transduction system. The IFN-*γ*-modified MSCs inhibit tumor cell growth* in vitro* and* in vivo* through a TRAIL-mediated pathway. Thus, IFN-*γ*-modified MSCs may provide a new option for cancer therapy.

## Supplementary Material

After fixation and co-culture with fluorescence-labeled antibodies against CD29, CD34, CD44, CD45, CD73, CD90, CD105 and CD166 (BD Pharmingen), respectively, the immunophenotypes of MSCs and MSCs IFN-*γ* were analyzed by flow cytometry..
The adipogenic and osteogenic differentiation of MSCs and MSCs IFN-*γ* were performed as described in Materials and Methods. At the end of induction, the differentiation capability of MSCs and MSCs IFN-*γ* were identified by oil red O and alizarin red S staining, respectively.
The same quantity (5x104/well) of MSCs and MSCs IFN-*γ* were plated and cultured for 48 h. at last, the cell number were measured by CCK-8 kit.
