# Supplementary material for: IFN-γ-Secreting-Mesenchymal Stem Cells Exert an Antitumor Effect In Vivo via the TRAIL Pathway
Source: J Immunol Res. 2014 May 26;2014:318098. doi: 10.1155/2014/318098 (PMC4058226; doi:10.1155/2014/318098)
Supplement: Supplementary file 1 — After fixation and co-culture with fluorescence-labeled antibodies against CD29, CD34, CD44, CD45, CD73, CD90, CD105 and CD166 (BD Pharmingen), respectively, the immunophenotypes of MSCs and MSCs IFN-γ were analyzed by flow cytometry.. The adipogenic and osteogenic differentiation of MSCs and MSCs IFN-γ were performed as described in Materials and Methods. At the end of induction, the differentiation capability of MSCs and MSCs IFN-γ were identified by oil red O and alizarin red S staining, respectively. The same quantity (5x10^4^/well) of MSCs and MSCs IFN-γ were plated and cultured for 48 h. at last, the cell number were measured by CCK-8 kit. [file 318098.f1.pdf]

Figure S1 The immunophenotype, differentiation potential and proliferation property between MSCs and MSCs IFN-γ were compared. (A) The immunophenotype of hMSCs and MSCs IFN-γ were analyzed by FACS. (B) The adipogenic and Osteogenic differentiation of MSCs and MSCs IFN-γ were assessed by oil red O staining and alizarin red S staining, respectively. (Magnification X200). (C) The proliferation potential of the same quantity of MSCs and MSCs IFN-γ within 48 h were evaluated by CCK-8 kit.

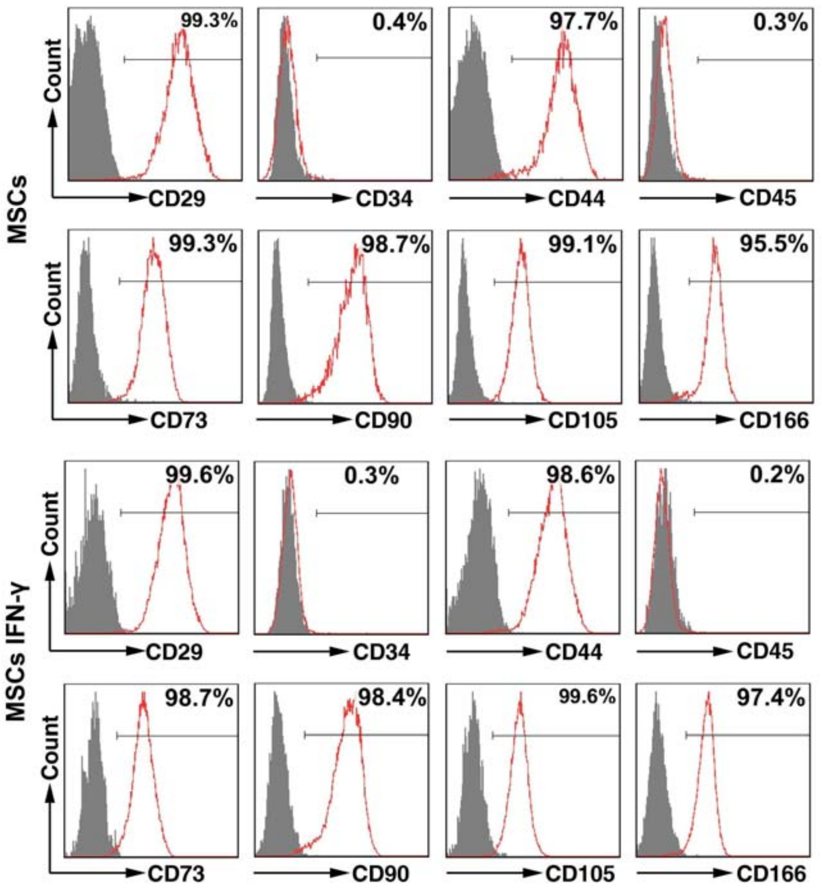
MSCs
Count
99.3%
0.4%
97.7%
0.3%
CD29
CD34
CD44
CD45
99.3%
98.7%
99.1%
95.5%
CD73
CD90
CD105
CD166
MSCs IFN-γ
99.6%
0.3%
98.6%
0.2%
CD29
CD34
CD44
CD45
98.7%
98.4%
99.6%
97.4%
CD73
CD90
CD105
CD166

(a)

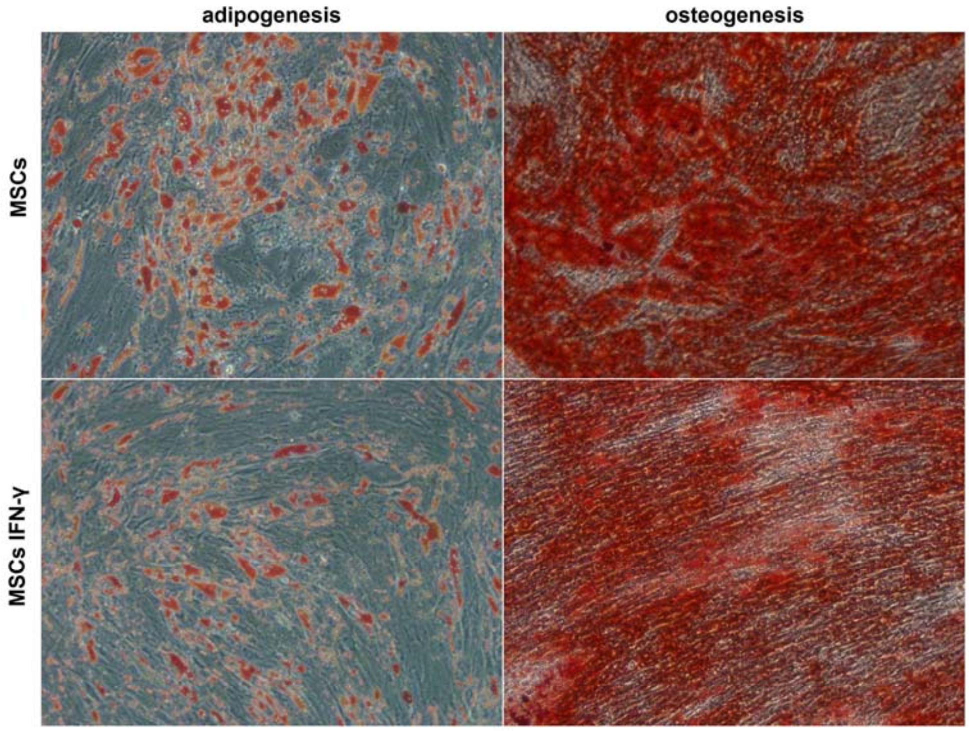
adipogenesis
osteogenesis
MSCs
MSCs IFN-γ

(b)

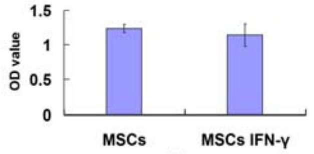
OD value
1.5
1
0.5
0
MSCs
MSCs IFN-γ

(c)
